# Sociodemographic and Service Use Characteristics of Abortion Fund Cases from Six States in the U.S. Southeast

**DOI:** 10.3390/ijerph18073813

**Published:** 2021-04-06

**Authors:** Whitney S. Rice, Katie Labgold, Quita Tinsley Peterson, Megan Higdon, Oriaku Njoku

**Affiliations:** 1 Department of Behavioral, Social and Health Education Sciences, Rollins School of Public Health, Emory University, Atlanta, GA 30322, USA; 2 The Center for Reproductive Health Research in the Southeast, Rollins School of Public Health, Emory University, Atlanta, GA 30030, USA; catherine.anne.labgold@emory.edu; 3 Department of Epidemiology, Rollins School of Public Health, Emory University, Atlanta, GA 30322, USA; 4 Access Reproductive Care–Southeast, Atlanta, GA 30357, USA; volunteer@arc-southeast.org (Q.T.P.); info@arc-southeast.org (O.N.); 5 Independent Researcher, Atlanta, GA 30327, USA; meganhigdon@gmail.com

**Keywords:** reproductive justice, pregnancy, abortion, pregnancy termination, sexual and reproductive health, family planning, access to health, U.S. state laws, policy

## Abstract

Abortion funds are key actors in mitigating barriers to abortion access, particularly in contexts where state-level abortion access restrictions are concentrated. Using 2017–2019 case management data from a regional abortion fund in the southeastern U.S., we described the sociodemographic and service use characteristics of cases overall (*n* = 9585) and stratified by state of residence (Alabama, Florida, Georgia, Mississippi, South Carolina, and Tennessee). Overall, cases represented people seeking abortion fund assistance who predominately identified as non-Hispanic Black (81%), 18–34 years of age (84%), publicly or uninsured (87%), having completed a high school degree or some college (70%), having one or more children (77%), and as Christian (58%). Most cases involved an in-state clinic (81%), clinic travel distance under 50 miles (63%), surgical abortion (66%), and pregnancy under 13 weeks’ gestation (73%), with variation across states. The median abortion fund contribution pledge was $75 (interquartile range (IQR): 60–100), supplementing median caller contributions of $200 (IQR: 40–300). These data provide a unique snapshot of a population navigating disproportionate, intersecting barriers to abortion access, and abortion fund capacity for social care and science. Findings can inform abortion fund development, data quality improvement efforts, as well as reproductive health, rights and justice advocacy, policy, and research.

## 1. Introduction

Over the past decade, state-level policies more frequently and repeatedly restricted abortion access in the United States (U.S.), compared to the prior decades since Roe v. Wade established the legal right to abortion [1]. Restrictive abortion policies, such as bans on insurance coverage, targeted regulation of abortion providers, gestational age limits, and medication abortion restrictions, are heavily concentrated in a few geographic regions [2]. The southeastern U.S. geographic region, in particular, has served as a legislative battleground for disputes over increasingly extreme state attempts to ban abortion [3], including cases elevated to federal courts and the U.S. Supreme Court [4]. In this way, southeastern U.S. states have been a central focus of national attention, as local abortion policy decisions have implications for abortion access in other states and regions.

Considering state laws, constitutions and court decisions surrounding abortion, each of the southeastern U.S. states, with the exception of Florida, are designated as hostile to abortion rights by the Center for Reproductive Rights [5]. Catalyzed by this policy context, within the greater historical context of the Deep South, the U.S. Southeast is a hub for grassroots activism, social justice organizing, and community support to advance reproductive justice [6]. Reproductive justice is a Black feminist theory, practice, and praxis grounded in the rights for all people to determine if and when to become pregnant, to prevent or end pregnancy, to parent in healthy, safe and supportive environments, and to healthy sexuality and the conditions in which to do so [7].

Consistent with the patterns of health disadvantage created and maintained by other historically and systemically oppressive policies and practices (e.g., slavery, Jim Crow laws, coerced sterilization, Medicaid non-expansion) [8,9,10,11], abortion policies share the potential to worsen intersectional health and social inequities, particularly among Black, Indigenous and socioeconomically vulnerable people in the U.S. Southeast. For example, state lawmakers often justify abortion restrictions as for the benefit of “unborn” or “early” children and infants [12]. Paradoxically, several studies suggest that U.S. states with greater government-sanctioned barriers to abortion access share characteristics less conducive to maternal and child health including reduced social supports and adverse infant health outcomes [13,14]. A 2020 analysis including all U.S. southeastern states found that states with more enacted restrictive abortion policies had fewer policies in place in support of pregnancy, women, and families [15]. A recent study found that increased U.S. state-level abortion policy restrictions were associated with higher probability of preterm birth and low birthweight among Black and less educated birthing people in the U.S. from 2005 to 2015 [2].

The state-level policy climate further shapes and exacerbates barriers to person-centered abortion access in the U.S. South. Multiple studies highlight logistical and financial barriers imposed by requirements to travel to an abortion clinic for a consultation visit followed by a waiting period prior to accessing services. A study of women accessing two abortion facilities in Alabama indicated that 42% of women traveled 25 or more miles for care, with longer travel distances (50–100 miles) associated with longer intervals between visits [16]. A second qualitative study of Louisiana residents seeking abortion care at the in-state facilities found that half of the participants lived over 30 min from the nearest facility, and inability to access care at convenient times contributed to the potential for care delay or nonreceipt [17].

The lack of abortion coverage by Medicaid creates additional barriers for abortion access. A Louisiana study of women seeking prenatal care found that a substantial proportion of people could not obtain an abortion due to Medicaid coverage restrictions [18]. The financial barriers to abortion access have been further documented by the Turnaway Study, which found that most women who received an abortion at one of 30 clinical facilities across the U.S. paid out-of-pocket costs for abortion, ranging from $0–$3700 (mean: $474) [19]. Costs varied by gestational age, abortion type, and financial assistance (none or provided by insurance, Medicaid, or other organizations). Most of the participants in this study reported that difficulty with raising money for the abortion resulted in delay in obtaining an abortion. In the Turnaway Study, 29% of participants received financial assistance to cover the cost of an abortion from organizations other than private insurance or Medicaid [19]. Women who received financial assistance from other organizations paid less out of pocket costs compared to those with no financial support.

Abortion funds—i.e., grassroots organizations that help provide financial support to persons seeking abortion services—play an essential role in mitigating the financial and logistical barriers to accessing abortion care [20]. Services offered vary across abortion funds, and include funds for abortion care, travel, lodging, childcare, doula, and translation services [21]. In the U.S., the National Network of Abortion Funds (NNAF), a membership organization that seeks to unify and build power among abortion fund clients, volunteers, donors and staff through person-centered and racial, economic and reproductive justice-based approaches, includes approximately 70 abortion fund organizations in 38 U.S. states and three countries. Although financial and logistical barriers to abortion access have been well-documented in the literature, our scientific understanding of the role of abortion funds in abortion access is limited.

Few studies have characterized U.S. abortion fund case management data, which can provide important insights into abortion access patterns and gaps. Studies from the 1980s and 90s of the North Carolina State Abortion Fund evaluated the association of fund support with local pregnancy outcomes, reporting that reduced abortion fund support resulted in fewer abortions and increased births [22,23]. Another study analyzed data from qualitative interviews with women to learn about their experiences following referral by three Massachusetts abortion funds to a state-subsidized insurance program for abortion care in 2010 [24]. Other studies described the sociodemographic and service use characteristics of clients of the countrywide NNAF Tiller Memorial Fund from 2010–2015 [25,26,27,28], which has since disbanded, with funds now redistributed to local NNAF member organizations. More recently, demographic and service use characteristics of recipients of financial pledges from a Florida abortion fund from 2001–2015 were published [29]. Finally, a qualitative case study explored abortion-related financial needs, physical safety needs, and abortion fund service characteristics from a review of 2017 case records from West Fund in Texas [30].

Given the role of abortion funds in providing a key social safety net for abortion care, analyses of abortion fund case management data are valuable for evaluating inequities in abortion access. A greater understanding of who is seeking abortion fund support at the state-level is needed to better inform resource investment in abortion funds, and abortion access policy, advocacy, organizing, and research. The current analysis seeks to fill research gaps in understanding of the population of cases managed by a multi-state abortion fund serving the southeastern U.S.

## 2. Materials and Methods

### 2.1. Study Population

Access Reproductive Care-Southeast (ARC-Southeast) is a regional reproductive justice non-profit and NNAF member organization based in Atlanta, GA that provides funding and logistical support to ensure safe and compassionate reproductive health care, including abortion services, to people living in Alabama, Florida, Georgia, Mississippi, South Carolina, and Tennessee. ARC-Southeast provides abortion funds and practical support (i.e., childcare, travel, and other logistical needs) following requests via phone or online, pending availability of funds and requestor residence within the above six states. Individuals seeking abortion funds or practical support become connected with ARC-Southeast through a variety of mechanisms including online searches for abortion fund services, word of mouth, referral from clinics and other abortion funds, and the National Abortion Federation (NAF) hotline. We refer to individuals seeking support from ARC-Southeast as “callers”, regardless of their original mode of contact, given that all individuals seeking support eventually interact with ARC-Southeast by phone as part of the intake process.

### 2.2. Data Source

We analyzed administrative data collected by ARC-Southeast as part of their abortion fund case management system, from 1 January 2017 through 31 December 2019. ARC-Southeast has a standardized data collection process whereby everyone who contacts ARC-Southeast as a caller, donor, volunteer, clinic partner, or other reproductive health, rights, and justice organization has a unique contact record within their database. Individuals seeking support for abortion access are distinguished from other contacts by the creation of a case record. A separate case is created for each procedure; thus, a single person can have several case files.

Sociodemographic and abortion fund support characteristics are collected by trained staff members via phone. Particularly when requests are initiated online via the ARC-Southeast website and when callers are unable to complete intake during an initial call, follow-up intake phone calls are conducted, as needed. Trained staff members collect information in the following order: required contact information, optional sociodemographic information, and case information. Required contact information includes name, case number, date of birth (age), phone number/email, and ZIP code of residence. Optional sociodemographic information collected by ARC-Southeast includes caller self-identified race and ethnicity, languages, education, country of birth, religious affiliation, and number of children. ARC-Southeast staff uses the following script to share with callers that the sociodemographic information is used for internal development and advocacy: “Ok, now we are moving on to the optional demographic information. This has no impact on potential funding, but it does help us do more targeted outreach in the South and help us do work to eliminate barriers to abortion access. Would you like to answer those questions?”

Lastly, case record information (which we refer to as service use characteristics) includes the gestational age at clinic appointment, abortion clinic name and location, total cost of abortion services and practical support needs. Case record information also includes abortion care payment information. Abortion fund pledges are provided by ARC-Southeast on a case-by-case basis after callers have attempted to obtain financial support from NAF. Callers are eligible for support from ARC-Southeast if they live in one of the six states served by ARC-Southeast and there are funds available for distribution. Pledges are paid to clinics after a caller obtains abortion services and provides ARC-Southeast with a service invoice. Accordingly, case records document the dollar amounts contributed to the cost of abortion care that were pledged by ARC-Southeast, contributed by other payment sources (i.e., NAF, public or private insurance, etc.), and paid by the caller.

### 2.3. Analyses

Although ARC-Southeast opens a case for every call or online request, we restricted the present analysis to cases eligible for support. We also excluded from the study population cases without valid geographic information on both the ZIP code of residence and the abortion clinic. We calculated the proportion of cases for which callers visited a clinic in their state of residence as the number of cases from callers who attended a clinic in a state matching the reported state of residence, divided by the total number of cases from that same state. To calculate the distance traveled to the clinic, we used the R package ‘osrm’, which provides an interface for calculating drive distance and time matrices using OpenStreetMaps data [31]. Distances were calculated from the case ZIP code centroid latitude and longitude to the clinic latitude and longitude.

We described the sociodemographic and service use characteristics of ARC-Southeast cases overall and stratified by each of the six states where ARC-Southeast provides support. We present the analysis of cases rather than callers, since it is possible for a single caller to have multiple cases over time. Potential repeat callers were identified based on name, residence, race/ethnicity, and similar age information. Details on the categorization of race and ethnicity, and country of birth for this analysis are described in Table 1 and Table 2.

To evaluate differences between states for key characteristics, we compared the mean and standard deviation for continuous covariates and the counted and proportion of total cases for categorical covariates. For financial characteristics, we calculated the median dollars and the interquartile range (25th and 75th percentiles). Given the goal of this analysis (to describe the entire population of ARC-Southeast callers during the study period), we did not statistically test for differences between states. We conducted two sensitivity analyses to assess missing geographic data in the sociodemographic and clinical characteristic distributions of ARC-Southeast cases. We first investigated missing geographic data by comparing the sociodemographic variables for cases with and without geographic information. We further described missingness by sociodemographic and clinic characteristics stratified by state of residence.

## 3. Results

ARC-Southeast opened 12,403 case records from 1 January 2017 through 31 December 2019, of which 10,172 (82%) represent callers living in Alabama, Florida, Georgia, Mississippi, South Carolina, or Tennessee. Geographic information was missing for 542 (5%) cases, thus our final sample included 9585 case records. In total, 332 case records (4%) were identified as potential repeat callers. Cases managed by ARC-Southeast increased substantially over time for all states (Table 3). Over half of total cases for the study period were opened during 2019 in all states except Alabama.

Sociodemographic characteristics for ARC-Southeast cases did not vary between states for the most part. The persons represented in ARC-Southeast cases predominately identified as non-Hispanic Black race and ethnicity, were between 18 and 34 years of age, had a high school degree or some college education, had one or two children, and were born in the U.S. Notably, although five racial and ethnic categories and two country of birth categories are represented in Table 3, more nuanced detail regarding how ARC-Southeast callers categorized themselves is provided in Table 1 and Table 2. ARC-Southeast callers represented 141 unique racial and ethnic self-identifications and 66 unique international countries of birth. For all states, at least half of cases represented individuals who identified as religious (and predominantly Christian). Cases were largely publicly insured or uninsured, though insurance status varied by case state of residence. Specifically, a larger proportion of uninsured callers were Mississippi residents (60%) compared to residents of other states (range: 33–45%).

Across all states, the majority of cases represented callers with a gestational age at appointment between 0–10 weeks (64%), and who obtained a surgical abortion (65%). Moreover, 48% of cases represented individuals who resided 0–24 miles from the abortion clinic that they visited for care, but a notable proportion resided greater than 100 miles from the clinic (23%). In comparison to sociodemographic characteristics, service use characteristics of abortion cases and funding varied to a greater degree between states of residence (Table 4). Abortion type varied by state of residence. Mississippi had a larger proportion of cases involving medication abortion (56%) as compared to other states (range: 25–39%).

With respect to funding, ARC-Southeast documented pledges to about 80% of all cases across states, except for Alabama (59%). Further, there were no meaningful differences in the median and interquartile range (IQR) for ARC-Southeast abortion fund pledge amounts across states. However, caller contributions varied to a greater degree between states, with cases in Florida ($220) and Georgia ($200) having greater caller contributions than other states (range: $150–175).

The proportion of cases where callers attended a clinic in their state of residence varied by state of residence. Over 90% of cases for Georgia or Florida residents involved attendance at a clinic in the same state of residence, in contrast to 51% and 18% of cases in Alabama and South Carolina, respectively. Lastly, travel distance to clinic further varied by state. The majority of cases in Florida (73%), Georgia (54%), and Tennessee (64%) accessed clinics within 25 miles of their zip code of residence. Alternatively, only 26% of Alabama cases, 33% of Mississippi cases, and 14% of South Carolina cases were within 25 miles. In these three states, a substantial proportion of cases (32–46%) traveled greater than 100 miles to a clinic for abortion care.

**Figure 1:**
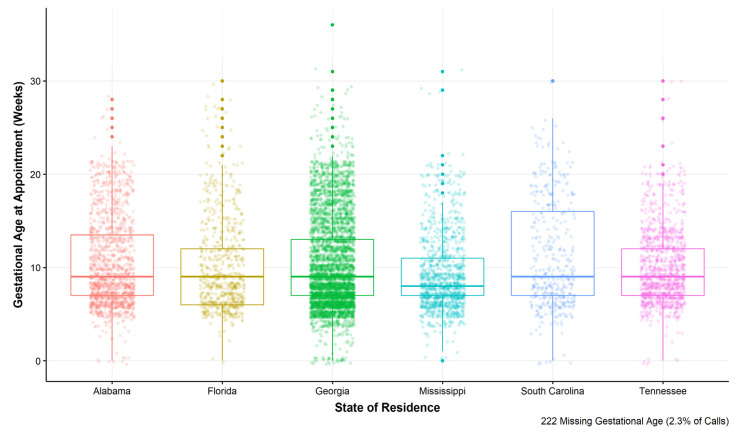
Variation in gestational age at appointment by state of residence for cases representing callers residing in the six southeastern states where ARC-Southeast provides assistance, 1 January 2017 through 31 December 2019.

There were slight differences in the gestational age at appointment between states, with the greatest proportion of cases involving gestational age between 0–10 weeks in Mississippi (74%) and the lowest proportion of cases for callers residing in South Carolina (57%) (Table 4). To better highlight the variation in gestational age in weeks by ARC-Southeast case state of residence, we calculated a box plot, which provides the median and interquartile range of gestational age at appointment for each state (Figure 1). Despite similar median gestational age at appointment across states (8–9 weeks), there is variation in the interquartile range, the outliers, and the density of observations for each state.

**Figure 2:**
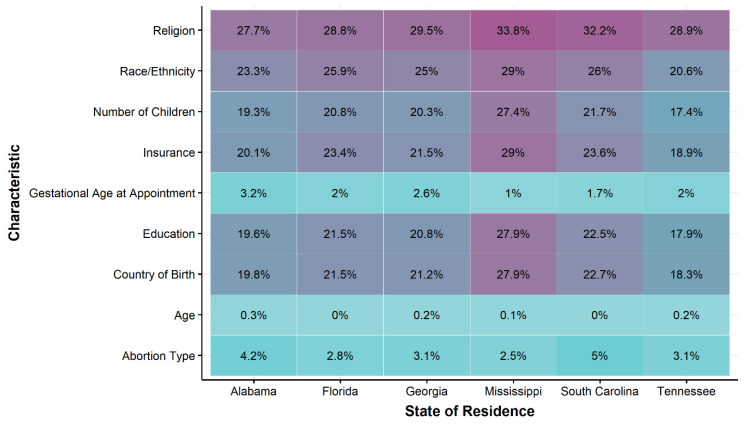
Percentage of missing data for sociodemographic and support characteristics for cases representing callers residing in the six southeastern states where Access Reproductive Care (ARC)-Southeast provides assistance, 1 January 2017 through 31 December 2019.

Across states of residence, service use characteristics had almost complete data (<5% missing), but sociodemographic characteristics of callers often included missing information, with several characteristics having greater than 20% missing data (e.g., country of birth, number of children, insurance status, race and ethnicity, and religion or faith) (Figure 2). For characteristics with a larger proportion of missing values (>20% missing), cases residing in the state of Mississippi had consistently greater proportion of missing observations per the total cases compared to other states (Figure 2). Further, the sensitivity analyses of missing data by geographic data availability suggested that there were differences in most demographic and service use characteristics between those with and without valid information for ZIP code of residence and the abortion clinic (Table 5). The proportion of cases opened in 2017 was greater for the missing geographic group (65%) compared to the valid geographic group (18%). Additionally, the proportion of cases funded was substantially larger in the valid geographic group (76%) compared to the missing geographic group (21%).

## 4. Discussion

The results of our study add to the scientific literature regarding individuals seeking financial and practical support for abortion care by describing the distribution of sociodemographic and service use characteristics for a large abortion fund in the southeast. This study joins a small group of prior analyses in advancing the body of evidence around the population seeking abortion fund support in the U.S. [22,23,24,25,26,27,28,29,30]. There are several strengths of the presented analyses, which begin to address important gaps in the literature. First, the volume of cases analyzed for this study (*n* = 9585) is substantially larger than that the analytic sample reported in prior national abortion fund studies, despite spanning fewer years of data and a regional sample. This study additionally provides a broader perspective around the variation in needs for abortion fund support by characterizing abortion fund cases beyond the case details reported in prior studies, including a state of residence-specific breakdown of a comprehensive set of sociodemographic and service use characteristics across multiple states. This analysis advances the existing literature by describing a large number of abortion fund callers’ educational attainment, religious affiliation, country of birth, abortion type, and distance traveled to abortion clinics.

The number of cases managed by ARC-Southeast grew over the study time period, likely reflecting ARC-Southeast’s organization growth. Cases from all states increased year-to-year, with the greatest proportion of cases over the study period opened in 2019, except for cases from Tennessee residents in 2018 and Alabama residents in 2019. These divergent patterns in cases over time could be related to change in available abortion fund resources in those states at those times. For example, increased visibility of and donations to the Alabama state-focused Yellowhammer Fund after the introduction of several 6-week abortion bans across the South in 2019 may have contributed to a lower number of cases opened at ARC-Southeast for Alabama residents in the same year [32].

Variation in the total number of ARC-Southeast callers by state may also reflect aspects of their organizational reach. Anecdotally, ARC-Southeast selected its location in Atlanta, Georgia in part because ARC-Southeast founders observed through work in abortion clinics that Atlanta is a regional hub for abortion care. Of the six southeastern states where ARC-Southeast provides assistance, most abortion clinics are located in Georgia [33]. As such, ARC-Southeast has the closest relationships with clinics is Georgia, and these clinics may be more likely to refer clients in need of financial or practical support to ARC-Southeast. Additionally, Alabama, Florida, and Mississippi are each home to local abortion funds [34]. The availability of funds from other sources may result in fewer callers from these states.

Characteristics of the population served by ARC-Southeast from 2017–2019 share similarities with the populations characterized in previous published analyses of national and state-specific abortion fund case data [26,27,30]. In particular, ARC-Southeast case data were comprised of callers who predominantly identify as non-Hispanic Black, as young adults (25–29 years of age), as having public or no insurance, and as having 1–2 children. Beyond the sociodemographic information available for previous abortion fund case studies, we found that the population served by ARC-Southeast largely had a high school degree or some college education, identified as religious (predominantly Christian), and were born in the U.S. Notably, inherent in ARC-Southeast’s holistic approach to sociodemographic data collection, this analysis was able to capture nuance and large variation within racial and ethnic self-categorization by abortion fund callers. More simplistic racial categorization alone often conflates race and ethnicity, masks within-group heterogeneity, and thus has implications for government policy, clinical practice and health outcomes [35,36,37]. Abortion funds can collect more nuanced racial and ethnic information, modeled after the data collection process used by ARC-Southeast, to explore the utility of culturally specific case management support (e.g., additional language support services) [38].

Another key difference from prior abortion fund case studies is that ARC-Southeast callers are largely in the first trimester of pregnancy, whereas over 70% of cases reported by studies from NNAF Tiller Memorial Fund were in the second trimester, which was a function of funding priorities for the Tiller Fund [39]. The distribution of ARC-Southeast cases by gestational age is more consistent with the distribution of abortions reported by the Centers for Disease Control and Prevention (CDC) for 2018, among which nearly all (92%) occurred in the first trimester-with variation by state and region [40]. The percentage of first trimester abortions in the 6 states served by ARC-Southeast ranged from 66–82 (mean: 73).

The data presented here are relevant to the evaluation of enacted state-level abortion access restrictions, and to ongoing legal battles surrounding attempts to further restrict abortion access in many of these states [4,12]. State laws that place upper gestational age limits on abortion (i.e., banning the use of abortion after a certain number of weeks from a person’s last menstrual period, with some exceptions) could contribute to some of the variation in gestational age at the time of abortion fund support use by state seen within our data. Several gestational age policies passed during or prior to the study period, including a 24 week ban in Florida [41], 22 week bans in Alabama, Georgia, and South Carolina [16,42,43], and 20-week ban in Mississippi [44]. Notably, while the majority of abortions in the U.S. occur within the first 13 weeks of gestation [40], a national survey found that most people who have had a second trimester abortion would have preferred an earlier abortion [45]. People seeking abortion, particularly younger and lower income individuals, report delays due to factors including cost, travel, and other access barriers (i.e., mandatory pre-abortion service consultation and subsequent waiting periods) to obtaining an abortion [16,46].

Additionally, for some states (Alabama, Mississippi, South Carolina, and Tennessee) [47], laws requiring the clinician prescribing medication abortion to be physically in the presence of a patient may have had implications for variation in abortion type between callers’ states of residence. Other possible explanations include differences in the number of clinics available to callers in each state (e.g., Mississippi only has one clinics), differences in clinic practices, and differences in patient population characteristics. Future research can leverage abortion fund or clinic administrative data and qualitative data to better understand reasons for the variation in these service use characteristics.

All of the states represented in this analysis also restrict abortion access by requiring that abortions be performed by a licensed physician, limiting use of public funding to pay for abortion services, and requiring parental consent for minors to have an abortion [48]. Each state has also passed laws requiring people seeking abortion to wait 24–48 h between required counseling and abortion care visits, with enjoined 24- and 48-h waiting periods in Florida and Tennessee respectively. We found that ARC-Southeast provided a median of $75 dollars to offset abortion service costs, while callers contributed a median of $200. Additionally, a little over half of the sample traveled between 25–300+ miles to an abortion clinic for services, with 19% of cases on average traveling out of state for services-and wide variation in that proportion by state (range: 4–82%). These data highlight that many people in these six Southeast states had unmet needs for financial and logistical support for abortion services, mitigated in part by the support provided by ARC-Southeast.

### Limitations

The study results should be considered in the context of several limitations. First, this analysis was cross-sectional and relied on administrative records that were not collected for research purposes. Thus, the present reporting of characteristics can provide an understanding of the population of individuals seeking services at ARC-Southeast and identify gaps in access. However, results should not be used to explain differences between states nor to describe the underlying population of individuals seeking abortions in these states without consideration of sampling bias. Additionally, there was an inability to conclusively identify all repeat callers. Thus, data is reported in terms of cases due to the possibility of duplicate caller interaction.

The exploration of the proportion of missing data for caller characteristics stratified by state of residence and geographic data availability indicates that the data were likely not missing at random. We observed a low proportion of missingness across states (<5%) for required caller contact and service use characteristics, which was expected given the necessity of this information to provide funding support. We noted a relatively higher proportion of missingness (>20%) for several optional sociodemographic characteristic covariates, with variation by state. Team members at ARC-Southeast shared that the organization has improved their data collection over time, resulting in reductions to the proportion of missingness over time. Missing values remained for several optional sociodemographic variables in the most recent year. State-specific patterns in missing data may indicate geographic differences in level of comfort with sharing personal information.

Follow-up studies are needed to better understand this variation. The sensitivity analysis of characteristics by geographic data availability further supports that ARC-Southeast’s data collection has improved over time, with a larger proportion of missing geographic information in the earlier study years. The comparison of the proportion of cases with and without geographic information that received funding pledges supports ARC-Southeast’s descriptions of trends in administrative data management, such that funded calls are more likely to have more complete information. This provides valuable information for beginning to understand the usefulness of abortion fund data to inform both the need and receipt of abortion services in the US Southeast. These data-specific findings can inform improved data collection, data completeness, and decision-making for abortion funds including and beyond ARC-Southeast. For future study purposes, use of methods to account for missing data (e.g., multiple imputation) should be considered.

## 5. Conclusions

In a time when access to legal abortion in the United States is under unprecedented threat, particularly in southeastern U.S. states, a sense of the sociodemographic and service use characteristics reported by abortion fund callers in this region is particularly timely. Limited published scientific description of people seeking abortion care and of the barriers faced while seeking abortion care is available in U.S. Southeast states relative to other regions. These data provide an important snapshot of a large number of people in the U.S. Southeast who faced financial and logistical barriers to accessing abortion care. Our research provides information regarding how this population self-identifies, the type of abortion care they received, at which gestations, and how far they had to travel for abortion care, among other lived experiences. This analysis provides further evidence of the fundamental role that abortion funds can play in producing research evidence around abortion access.

Abortion funds have historically maintained a key role in mitigating gaps in access to abortion care and have seen increased demand amidst increasing abortion restriction policies. These data highlight the growing capacity of ARC-Southeast to distribute abortion funds and other abortion care support in six states “hostile” to reproductive health, rights, and justice, as well as opportunities to better serve people seeking abortion fund support through data informed strategies. This analysis is the product of a partnership by leaders of an abortion fund and a research center. Therefore, this work provides a model for outcomes that such cross-organizational engagement of expertise and resources can produce. These data can inform the work of other abortion funds who seek to describe the populations that they serve to those who use or consider using their services, for internal understanding and quality improvement, and in conversation with donors and funding organizations. This work can additionally inform future research that similarly attempts to describe populations seeking abortion and gaps in abortion access using abortion fund case management or other data, plus research that seeks to characterize reproductive health, rights and justice in the U.S. and U.S. Southeast.

The population served by ARC-Southeast shares geographic and sociodemographic characteristics with populations disproportionately burdened by ongoing attempts to limit abortion access through policy. Thus, findings are also particularly useful to reproductive health, rights and justice advocacy organizations who leverage information about gaps in abortion access in southeastern U.S. states in their public communications, events and reporting. Finally, results may also be of interest to professionals who contribute to reproductive health policy processes (e.g., policymakers, attorneys, and other experts) by describing the current context surrounding abortion care access.

## Figures and Tables

**Table 1:** Self-identified and categorized race and ethnicity group.

Categorized Race and Ethnicity	Self-Identified Race and Ethnicity
Asian or Pacific Islander	Asian Indian/Indian (Asia), Asian Indian/Indian (Asia)/African American, Asian Indian/Indian (Asia)/African American/Hispanic/Puerto Rican, Asian Indian/Indian (Asia)/African American/White/Caucasian, Asian Indian/Indian (Asia)/Black, Asian Indian/Indian (Asia)/Black/White/Caucasian/“Mixed”, Asian Indian/Indian (Asia)/Hispanic, Asian Indian/Indian (Asia)/White/Caucasian, Asian Indian/Indian (Asia)/White/Caucasian/“Mixed”, Asian or Pacific Islander, Asian or Pacific Islander/African American, Asian or Pacific Islander/Asian Indian/Indian (Asia)/White/Caucasian, Asian or Pacific Islander/Hispanic, Asian or Pacific Islander/White/Caucasian
Hispanic	Hispanic, Hispanic/“Mixed”, Hispanic/“Mixed”/Native American, Hispanic/American/United States/Mexican, Hispanic/Colombian/Dominican, Hispanic/Filipino/Philippine, Hispanic/Guatemalan, Hispanic/Honduran, Hispanic/Mexican, Hispanic/Puerto Rican, Hispanic/White/Caucasian, Hispanic/White/Caucasian/“Mixed”, Hispanic/White/Caucasian/Other
Indigenous American	Native American/Egyptian, Native American, White Mountain Apache
Non-Hispanic Black	African American, African America/“Mixed”, African American/Amerindian/Indigene/Indio, African American/Black, African American/Black/“Mixed”, African American/Black/Hispanic, African American/Cherokee/Irish, African American/Creole, African American/German, African American/Haitian/Mexican, African American/Hispanic, African American/Hispanic/“Mixed”, African American/Italian/Native American, African American/Other, African American/White/Caucasian, African American/White/Caucasian/“Mixed”, African American/White/Caucasian/Hawaiian/Native Hawaiian/Native American, African American/White/Caucasian/Korean, African American/White/Caucasian/Mexican, African American/White/Caucasian/Mexican/Native American, Black, Black/“Mixed”, Black/“Mixed”/East Indian/Italian, Black/“Mixed”/Vietnamese, Black/American/United States, Black/American/United States/Ibo/Igbo/Nigerian, Black/Dominican, Black/Haitian, Black/Hispanic, Black/Hispanic/White/Caucasian, Black/Italian, Black/Jamaican, Black/Jamaican/West Indian, Black/Korean, Black/Mexican, Black/Native American, Black/Nigerian, Black/Other, Black/Puerto Rican, Black/Spaniard/Spanish, Black/Trinidadian/Tobagonian, Black/Vietnamese, Black/White/Caucasian, Black/White/Caucasian/“Mixed”, Black/White/Caucasian/Cherokee
Non-Hispanic White	White/Caucasian, White/Caucasian/“Mixed”, White/Caucasian/“Mixed”/Chinese, White/Caucasian/“Mixed”/Puerto Rican, White/Caucasian/Cheyenne, White/Caucasian/Native American, White/Caucasian/Other
Missing	Decline to State, Unknown, “Mixed”, Other, Race not specified (American/United States, American/United States/Cuban, Amerindian/Indigene/Indio, Arab/Arabic, Bangladeshi, Brazilian, Chinese, Colombian, Creole, Egyptian, English, Ethiopian, German/Puerto Rican, Haitian, Hawaiian/Native Hawaiian/Spaniard/Spanish, Honduran, Indonesian, Irish, Italian, Jamaican, Jewish/Jew, Kenyan, Lao/Laotian/Portuguese, Latin American Indian, Liberian, Mexican, Nigerian, Other/Nigerian, Pakistani, Persian, Portuguese, Romanian, Thai, Vietnamese, West Indian)

**Table 2:** International countries of birth.

Categorized Country of Birth	Self-Identified Country of Birth
International	Afghanistan, Antigua and Barbuda, The Bahamas, Benin, Bhutan, Bosnia and Herzegovina, Brazil, Burundi, Cambodia, Cameroon, Canada, Chile, China, Colombia, Democratic Republic of the Congo, Cuba, Dominican Republic, El Salvador, Eritrea, Ethiopia, France, Georgia, Germany, Ghana, Guatemala, Guinea, Guyana, Haiti, Honduras, India, Iran, Italy, Jamaica, Jordan, Kenya, South Korea, Kuwait, Liberia, Mexico, Morocco, Namibia, Nicaragua, Nigeria, Pakistan, Panama, Peru, Philippines, Rwanda, Saint Kitts and Nevis, Saint Lucia, Samoa, Senegal, Seychelles, Sierra Leone, Somalia, South Africa, Spain, Thailand, Trinidad and Tobago, Turkey, Ukraine, United Kingdom, Vanuatu, Venezuela, Vietnam, Zambia
United States	United States

**Table 3:** Sociodemographic characteristics of abortion fund cases ^1^ representing callers residing in the six southeastern states where Access Reproductive Care (ARC)-Southeast provides assistance, 1 January 2017–31 December 2019 ^2^.

*n* (Column %) ^3^	Overall (*n* = 9585)	Alabama (*n* = 1421)	Florida (*n* = 715)	Georgia (*n* = 4637)	Mississippi (*n* = 1206)	South Carolina (*n* = 423)	Tennessee (*n* = 1183)
Year							
2017	1766 (18%)	402 (28%)	106 (15%)	824 (18%)	89 (7%)	67 (16%)	278 (23%)
2018	2534 (27%)	519 (37%)	175 (24%)	1279 (27%)	239 (20%)	77 (18%)	245 (21%)
2019	5285 (55%)	500 (35%)	434 (61%)	2534 (55%)	878 (73%)	279 (66%)	660 (56%)
Race/Ethnicity ^4^							
Asian or Pacific Islander	47 (1%)	----	----	28 (1%)	----	----	----
Hispanic	257 (4%)	18 (2%)	46 (9%)	150 (4%)	----	----	28 (3%)
Indigenous American	15 (<1%)	----	----	----	----	----	----
Non-Hispanic Black	5856 (81%)	873 (80%)	379 (72%)	2907 (84%)	754 (88%)	239 (76%)	704 (75%)
Non-Hispanic White	1033 (14%)	195 (18%)	100 (19%)	387 (11%)	90 (11%)	63 (20%)	198 (21%)
Missing	2377	331	185	1157	350	110	244
Age Category, Years							
<18	185 (2%)	24 (2%)	16 (2%)	70 (2%)	35 (3%)	----	30 (2%)
18–24	2504 (27%)	402 (28%)	199 (28%)	1232 (27%)	333 (27%)	109 (26%)	266 (23%)
25–29	3299 (35%)	483 (34%)	221 (31%)	1588 (34%)	411 (34%)	166 (39%)	430 (36%)
30–34	2225 (23%)	313 (22%)	190 (27%)	1044 (23%)	293 (24%)	93 (22%)	292 (25%)
35–39	977 (10%)	137 (10%)	73 (10%)	497 (10%)	114 (10%)	34 (8%)	122 (10%)
40+	342 (3%)	58 (4%)	16 (2%)	197 (4%)	19 (2%)	----	41 (4%)
Missing	16	4	0	9	1	0	2
Insurance Payor							
Private	954 (13%)	156 (14%)	67 (12%)	417 (11%)	118 (14%)	36 (11%)	160 (17%)
Public	3251 (43%)	500 (44%)	270 (49%)	1605 (44%)	223 (26%)	174 (54%)	479 (50%)
No Insurance	3257 (44%)	480 (42%)	211 (39%)	1618 (45%)	515 (60%)	113 (35%)	320 (33%)
Missing	2123	285	167	997	350	100	224
Highest Level of Education							
Less than high school	87 (1%)	----	----	42 (1%)	----	----	----
Some high school	853 (11%)	149 (13%)	69 (12%)	399 (11%)	68 (8%)	45 (14%)	123 (13%)
High school degree/GED	3097 (42%)	492 (43%)	283 (50%)	1487 (41%)	294 (34%)	144 (44%)	397 (41%)
Some College	2208 (29%)	333 (29%)	132 (24%)	1106 (30%)	280 (32%)	83 (25%)	274 (28%)
Trade/technical/vocational training	149 (2%)	----	----	77 (2%)	----	----	26 (3%)
College graduate or higher	1151 (15%)	147 (13%)	54 (10%)	562 (15%)	209 (24%)	42 (13%)	137 (14%)
Missing	2040	279	154	964	336	95	212
Religious Affiliation							
Christian	3914 (58%)	545 (53%)	292 (57%)	1900 (58%)	516 (65%)	171 (59%)	490 (58%)
Non-religious	1719 (26%)	262 (26%)	148 (29%)	859 (26%)	174 (22%)	63 (22%)	213 (25%)
Other	238 (3%)	30 (3%)	19 (4%)	116 (34%)	34 (4%)	17 (6%)	22 (3%)
Prefer Not To Answer	862 (13%)	190 (18%)	50 (10%)	396 (12%)	74 (9%)	36 (13%)	116 (14%)
Missing	2852	394	206	1366	408	136	342
Number of Children							
0	1747 (23%)	259 (23%)	99 (17%)	927 (25%)	186 (21%)	67 (20%)	209 (21%)
1–2	3963 (52%)	624 (54%)	303 (54%)	1848 (50%)	474 (54%)	191 (56%)	523 (54%)
3+	1884 (25%)	264 (23%)	164 (29%)	922 (25%)	216 (25%)	73 (22%)	245 (25%)
Missing	1991	274	149	940	330	92	206
Country of Birth							
United States	7295 (97%)	1131 (99%)	542 (97%)	3502 (96%)	867 (100%)	320 (98%)	933 (97%)
International ^5^	219 (3%)	---	19 (3%)	150 (4%)	---	---	33 (3%)
Missing	2071	282	154	985	337	96	217

^1^ A single caller can have more than one case during the study period.^2^ All cases with geographic information in the study period. ^3^ Categories with counts under 15 are censored as indicated by dashes. ^4^ Self-Identified race and ethnicity: Table 1. ^5^ Non-U.S. Country of Birth: Table 2.

**Table 4:** Abortion fund service use characteristics of abortion fund cases ^1^ representing callers residing in the six southeastern states where Access Reproductive Care (ARC)-Southeast provides assistance, 1 January 2017–31 December 2019 ^2^.

*n* (Column %) ^3^	Overall (*n* = 9585)	Alabama (*n* = 1421)	Florida (*n* = 715)	Georgia (*n* = 4637)	Mississippi (*n* = 1206)	South Carolina (*n* = 423)	Tennessee (*n* = 1183)
Gestational Age Category, Weeks							
0–10 weeks	5948 (64%)	836 (61%)	449 (64%)	2830 (63%)	884 (74%)	241 (58%)	708 (61%)
11–12 weeks	869 (9%)	124 (9%)	78 (11%)	378 (8%)	91 (8%)	36 (8%)	162 (14%)
13–15 weeks	1072 (11%)	169 (12%)	69 (10%)	503 (11%)	123 (10%)	33 (8%)	175 (15%)
16–18 weeks	744 (8%)	121 (9%)	49 (7%)	422 (9%)	46 (4%)	46 (11%)	60 (5%)
19–21 weeks	608 (7%)	114 (8%)	35 (5%)	322 (7%)	44 (4%)	44 (11%)	49 (4%)
22+ weeks	122 (1%)	----	21 (3%)	63 (2%)	----	16 (4%)	----
Missing	222	46	14	119	12	7	24
Abortion Type							
Medication Abortion	3202 (35%)	343 (25%)	272 (39%)	1434 (32%)	661 (56%)	127 (32%)	365 (32%)
Surgical Abortion	6072 (65%)	1018 (75%)	423 (61%)	3060 (68%)	515 (44%)	275 (68%)	781 (68%)
Missing	311	60	20	143	30	21	37
ARC-Southeast AF Pledge	7233 (76%)	834 (59%)	583 (82%)	3573 (77%)	973 (81%)	334 (79%)	936 (79%)
ARC-Southeast AF Pledge, Dollars ^4^	75 (60, 100)	85 (60, 100)	80 (60, 100)	75 (60, 100)	75 (60, 100)	85 (75, 125)	80 (75, 100)
ARC-Southeast AF Caller Contribution, Dollars ^3^	200 (40, 300)	150 (0, 275)	220 (100, 350)	200 (50, 300)	175 (29, 250)	165 (25, 274)	175 (0, 250)
Visiting Clinic in state of Residence	7652 (81%)	720 (51%)	652 (91%)	4366 (96%)	889 (74%)	75 (18%)	950 (81%)
Distance from Residential Zip Code to Clinic, Miles							
0–24 miles	4627 (48%)	377 (27%)	522 (73%)	2517 (54%)	394 (33%)	61 (14%)	756 (64%)
25–49 miles	1374 (14%)	179 (12%)	40 (6%)	922 (20%)	94 (8%)	59 (14%)	80 (7%)
50–99 miles	1386 (15%)	248 (18%)	30 (4%)	600 (13%)	321 (27%)	109 (26%)	78 (6%)
100–299 miles	1887 (20%)	566 (40%)	67 (9%)	527 (11%)	320 (26%)	163 (39%)	244 (21%)
300+ miles	311 (3%)	51 (4%)	56 (8%)	71 (2%)	77 (6%)	31 (7%)	25 (2%)

Abbreviations: AF = Abortion Fund, IQR = Interquartile range. ^1^ A single caller can have more than one case during the study period. ^2^ All cases with geographic information in the study period. ^3^ Categories with counts under 15 are censored as indicated by dashes. ^4^ Median (IQR).

**Table 5:** Comparison of abortion fund sociodemographic and support characteristics of abortion fund cases ^1^ for callers residing in the six southeastern states where Access Reproductive Care (ARC)-Southeast provides assistance, 1 January 2017–31 December 2019 for cases with and without valid geographic information on ZIP code of residence and abortion clinic.

*n* (Column %) ^2^	With Geographic Information	Without Geographic Information
	(*n* = 9585)	(*n* = 542)
Year		
2017	1766 (18%)	354 (65%)
2018	2534 (27%)	86 (16%)
2019	5285 (55%)	102 (19%)
State of Residence		
Alabama	1421 (15%)	65 (12%)
Florida	715 (8%)	39 (7%)
Georgia	4637 (48%)	319 (59%)
Mississippi	1206 (13%)	26 (5%)
South Carolina	423 (4%)	61 (11%)
Tennessee	1183 (12%)	32 (6%)
Race/Ethnicity ^3^		
Asian or Pacific Islander	47 (1%)	---
Hispanic	257 (4%)	15 (3%)
Indigenous American	15 (<1%)	---
Non-Hispanic Black	5856 (81%)	356 (80%)
Non-Hispanic White	1033 (14%)	67 (15%)
Missing	2377	95
Age Category, Years		
<18	185 (2%)	---
18–24	2504 (27%)	135 (25%)
25–29	3299 (35%)	185 (34%)
30–34	2225 (23%)	106 (20%)
35–39	977 (10%)	71 (13%)
40+	342 (3%)	28 (5%)
Missing	16	---
Insurance Payor		
Private	954 (13%)	43 (10%)
Public	3251 (43%)	221 (48%)
No Insurance	3257 (44%)	193 (42%)
Missing	2123	85
Highest Level of Education		
Less than high school	87 (1%)	---
Some high school	853 (11%)	65 (14%)
High school degree/GED	3097 (41%)	208 (45%)
Some College	2208 (29%)	118 (25%)
Trade/technical/vocational training	149 (2%)	---
College graduate or higher	1151 (15%)	66 (14%)
Missing	2040	75
Religious Affiliation		
Christian	3914 (58%)	232 (52%)
Non-religious	1719 (26%)	140 (32%)
Other	238 (3%)	20 (5%)
Prefer not to Answer	862 (13%)	43 (10%)
Missing	2852	107
Number of Children		
0	1747 (23%)	131 (28%)
1–2	3963 (52%)	225 (48%)
3+	1884 (25%)	111 (24%)
Missing	1991	75
Country of Birth		
United States	7295 (97%)	450 (97%)
International ^4^	219 (3%)	---
Missing	2071	80
Gestational Age Category, Weeks		
0–10 weeks	5948 (64%)	208 (55%)
11–12 weeks	869 (9%)	33 (9%)
13–15 weeks	1072 (11%)	40 (11%)
16–18 weeks	744 (8%)	36 (9.5)
19–21 weeks	608 (7%)	38 (10%)
22+ weeks	122 (1%)	23 (6%)
Missing	222	164
Abortion Type		
Medication Abortion	3202 (35%)	59 (17%)
Surgical Abortion	6072 (65%)	291 (83%)
Missing	311	192
ARC-Southeast AF Pledge	7233 (76%)	113 (21%)
Median ARC-Southeast AF Pledge, Dollars ^5^	75 (60, 100)	60 (50, 100)
Median ARC-Southeast AF Caller Contribution, Dollars ^5^	200 (40, 300)	0 (0,0)

Abbreviations: AF = Abortion Fund, IQR = Interquartile range. ^1^ A single caller can have more than one case during the study period. ^2^ Categories with counts under 15 are censored as indicated by dashes. ^3^ Self-Identified race and ethnicity: Table 1. ^4^ Non-US Country of Birth: Table 2. ^5^ Median (IQR).

## Data Availability

Data was obtained from ARC-Southeast and are not publicly available given that the data was originally collected for non-research purposes.
